# Supplementary material for: A new set of mutations in the second transmembrane helix of the Cox2p-W56R substantially improves its allotopic expression in Saccharomyces cerevisiae
Source: Genetics. 2025 Apr 3;229(4):iyaf037. doi: 10.1093/genetics/iyaf037 (PMC12005268; doi:10.1093/genetics/iyaf037)
Supplement: iyaf037_Supplementary_Data [file iyaf037_supplementary_data.zip › Supplementary_figures_legends.docx]

**Figure S1.** The obtained better growing epPCR clones were grown overnight at 30^∘^C in a 96 well plate along with the wild type (well A1), the *COX2* deletion strain (well A2) and the the *COX2* deletion strain expressing the sub-optimal *COX2-W56R* allotopic construct (well A3). Then, the strains were spotted on glucose rich media (left panel) or glycerol-containing rich media (right panel) and grown for 3 days at 30^∘^C and the colony size measured using a Phenobooth to generate the associated heatmaps where the pink means slow growth and the green denotes a fast growth (A). The epPCR clones were ranked based on the size of the formed colonies to isolate potential better growing strains (left chart) and the top nine better growers were subjected to a new spot test and grown 3 days at 30^∘^C to assess the growth benefit conferred by the mutations (B).

**Figure S2.** Hydropathy plots generated for each of the isolated epPCR clones using the ProtScale tool available on the ExPASy Server with the Kyte & Doolittle amino-acid scale and a window size of 7 (the y-axis corresponds to amino-acid hydrophobicity score and the x-axis the amino-acid position). For each plot, the dashed black line corresponds to the hydrophobic profile of the corresponding epPCR clone while the plain grey line corresponds to that of 1x MTS *OXA1*-*COX2-W56R* initial construct. The orange arrows denote a drop in hydrophobicity in TMH2 due to amino acid permutations in the hotspot of mutation while the green arrows highlight the drop of hydrophobicity in the MTS and IMS (clone F7).

**Figure S3.** Screening process to identify the most optimal allotopic expression conditions. (A) First, we compared the benefit of combining all the mutations identified in the epPCR clones to get the best ORF. (B) Then, we tested the benefit of swapping the promoter and modifying *OXA1* MTS copy number. (C) We monitored the growthin liquid culture of the different strains of interest in YPGly for 36h at 30^∘^C. (n=3, mean ± SD) (D) Finally, we completed the screening of the best expression conditions using the p*ICL1* promoter and comparing the benefit of adding 1 or 2 copies of OXA1 MTS or encoding the TU on a low- or high-copy vetor. For each spot test, the cells were grown for 4 days at 30^∘^C.

**Figure S4.** (A) Results of the Cox2p sequence comparison between *Saccharomyces cerevisiae* (UniProt ID: P00410) and *Homo sapiens* (UniProt ID: P00403) using the Clustal Omega tool available on the EMBL-EBI website. The amino acids are coloured using the Clustal colour code referring to their physico-chemical properties. The two rectangles are indicating the transmembrane domains. The arrows are indicating the position of the different adaptive mutations favouring Cox2p allotopic expression in the baker’s yeast (including the W56R substitution from Supekova *et al*., 2010). (B) Spot test presenting the growth benefit conferred by different human *COX2* allotopic constructs (codon-optimised for yeast) carrying potentially beneficial mutations identified in this study. The allotopic constructs were hosted on a low-copy plasmid, associated with one copy of the *OXA1* MTS, controlled by the *ICL1* promoter and incubated for 6 days at 30^∘^C. (C) Spot test presenting the growth benefit conferred by the different constructs previously described in Figure S6.B when combined with the leader peptide sequence from the yeast *COX2* and incubated for 6 days at 30^∘^C.
