# Supplementary figures and images for: A new set of mutations in the second transmembrane helix of the Cox2p-W56R substantially improves its allotopic expression in Saccharomyces cerevisiae
Source: Genetics. 2025 Apr 3;229(4):iyaf037. doi: 10.1093/genetics/iyaf037 (PMC12005268; doi:10.1093/genetics/iyaf037)

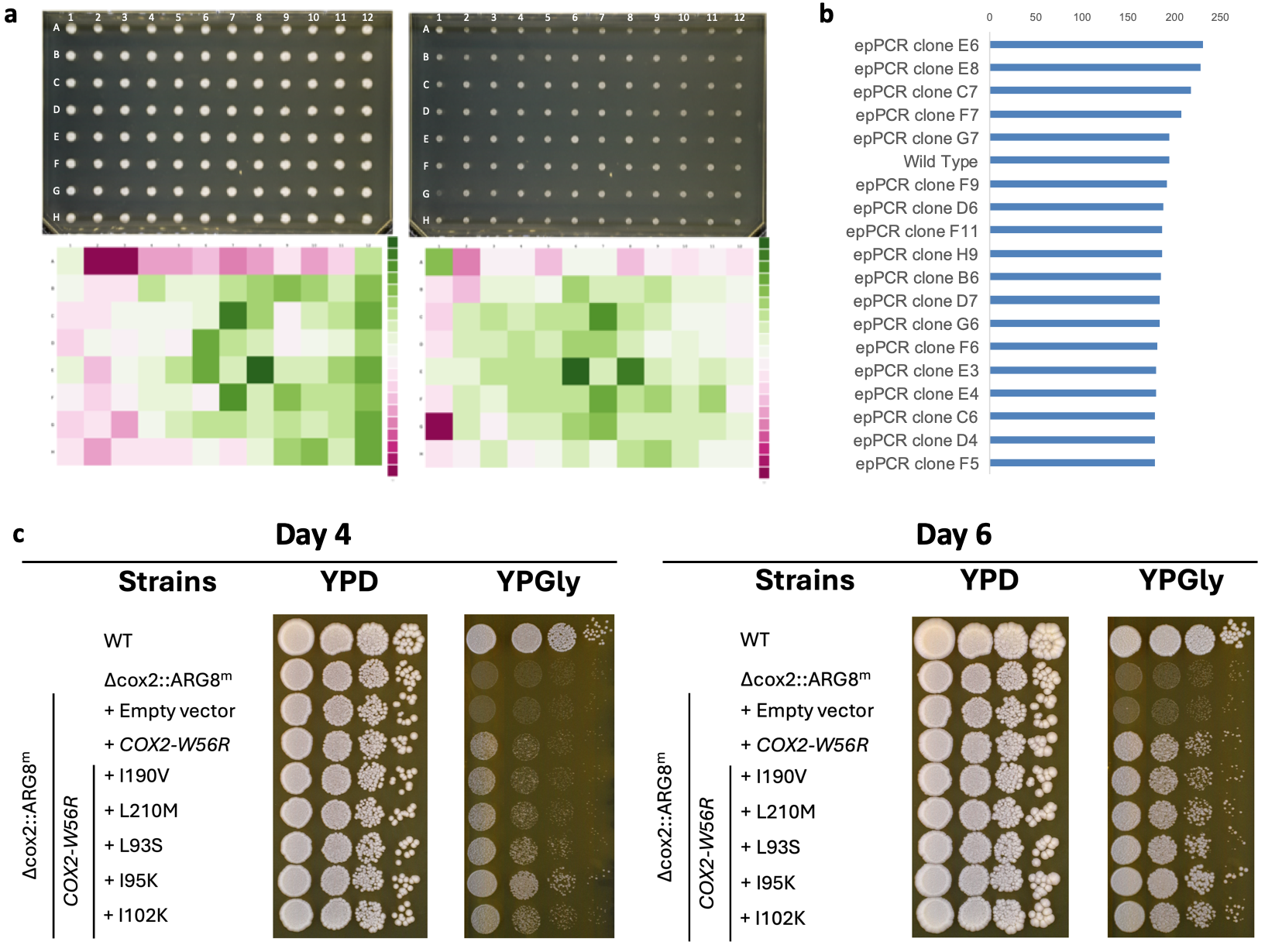

Supplement: iyaf037_Supplementary_Data [file iyaf037_supplementary_data.zip › Figure_S1_GENETICS-2024-307723.tif]

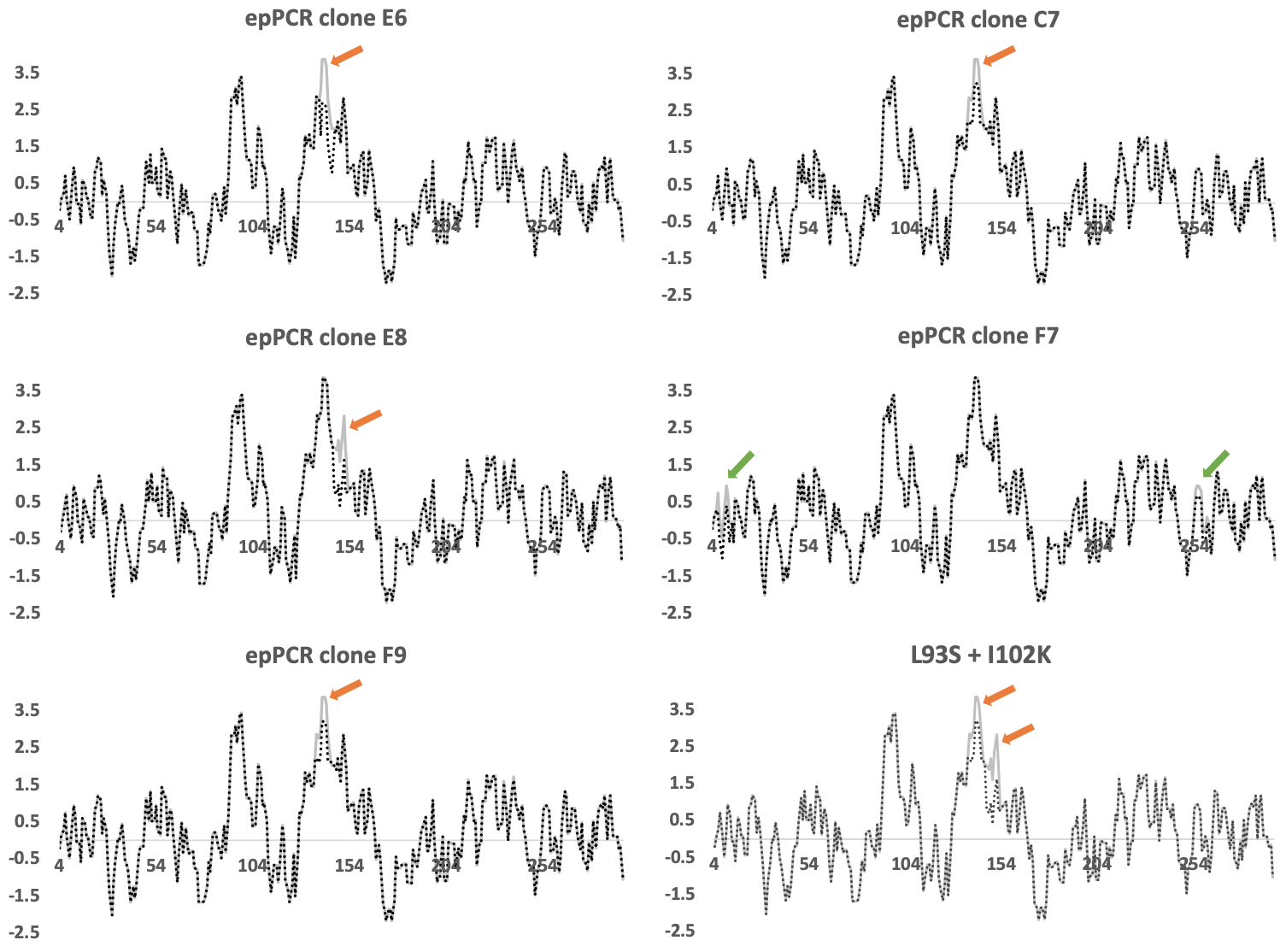

Supplement: iyaf037_Supplementary_Data [file iyaf037_supplementary_data.zip › Figure_S2_GENETICS-2024-307723.tif]

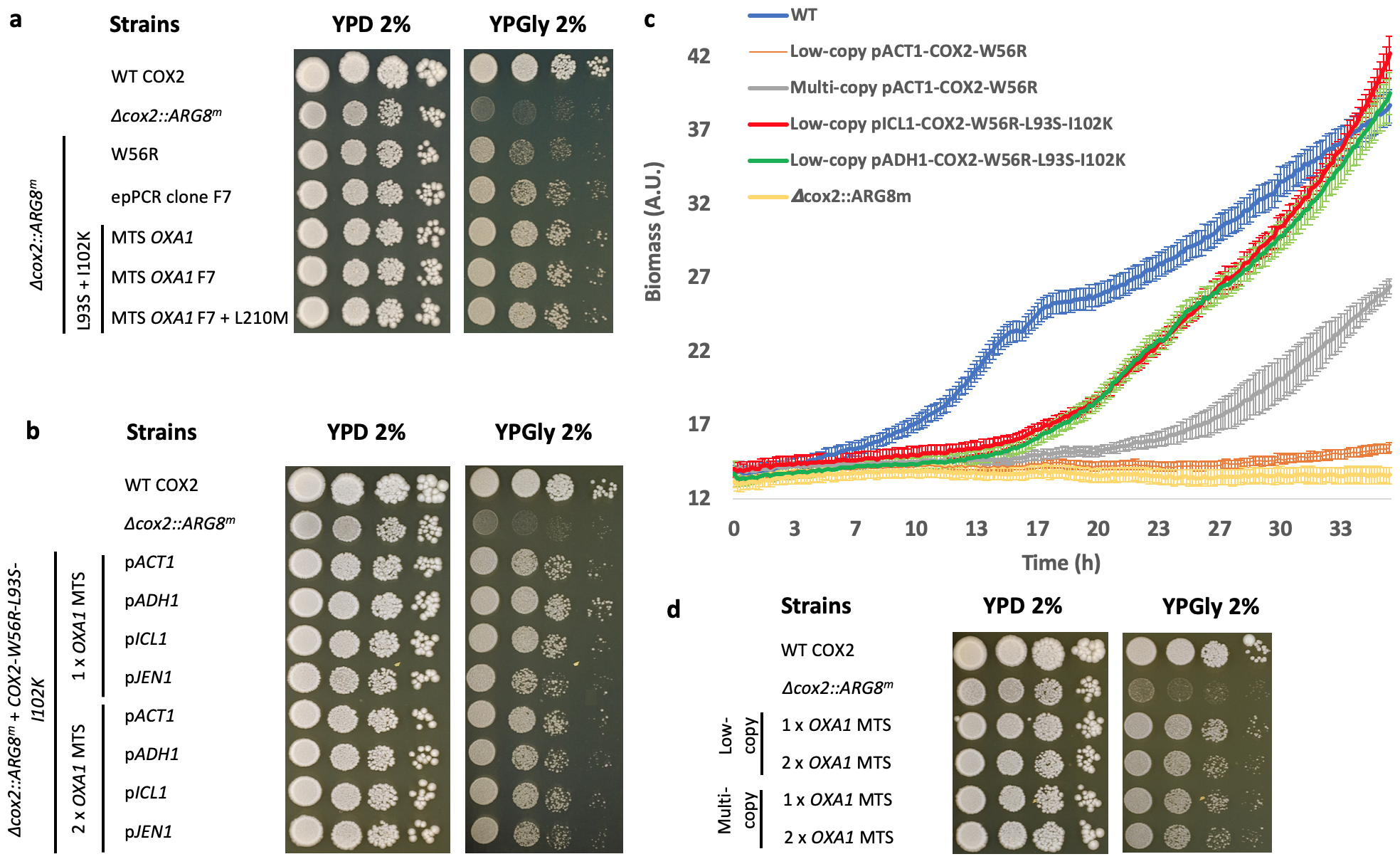

Supplement: iyaf037_Supplementary_Data [file iyaf037_supplementary_data.zip › Figure_S3_GENETICS-2024-307723.tif]

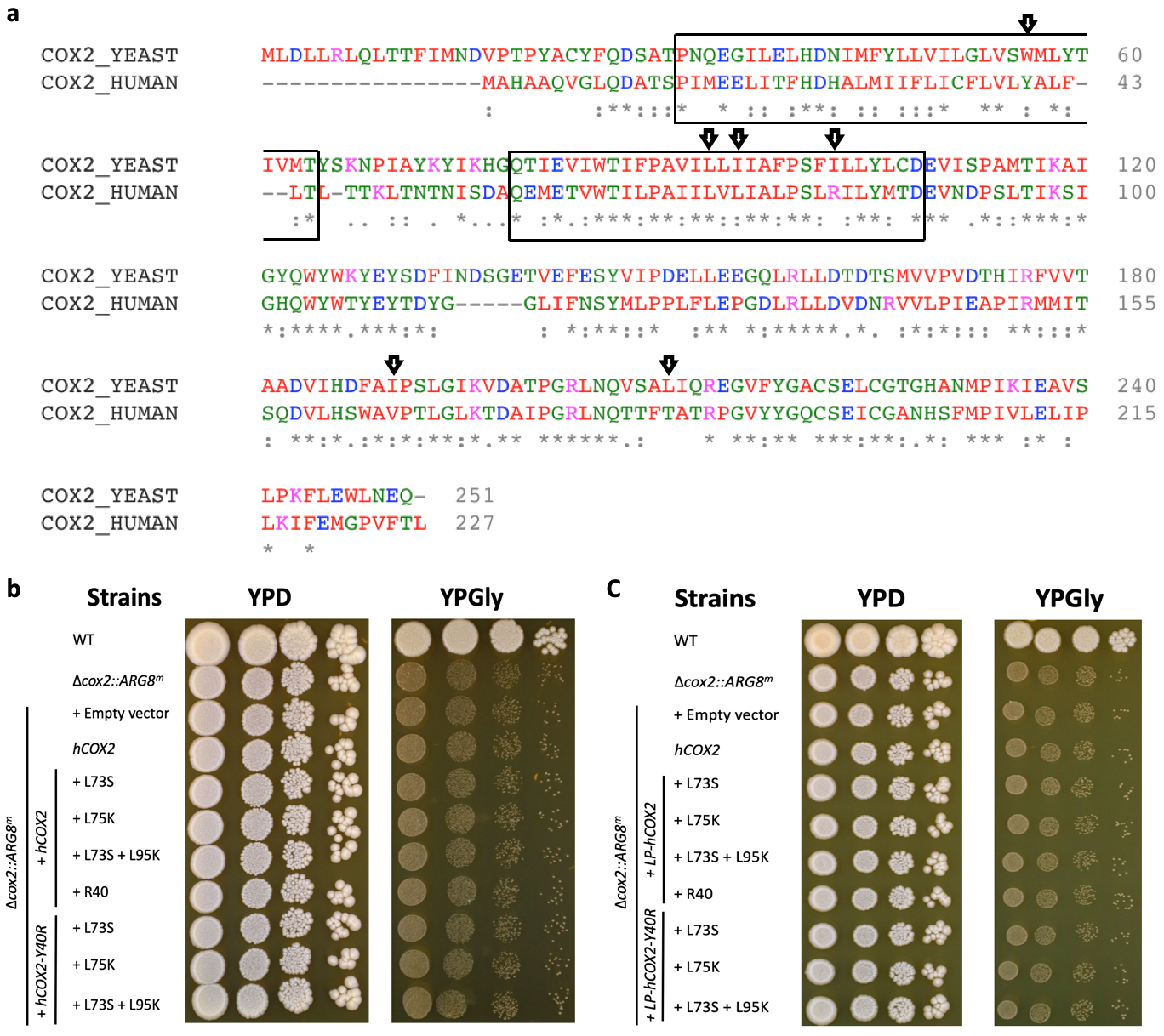

Supplement: iyaf037_Supplementary_Data [file iyaf037_supplementary_data.zip › Figure_S4_GENETICS-2024-307723.tif]
